# Prevalence of latent tuberculosis infection among healthcare workers in Saudi Arabia: a systematic review and meta-analysis

**DOI:** 10.1186/s12889-026-27751-0

**Published:** 2026-06-01

**Authors:** Yousef N. Alanazi, Afnan M. Alotaibi, Alaa Saeed Bahakam, Batool Awad Leslom, Raghad Ahmed Al Juhani, Abdalazez Mohammed Altayyar, Ahmad Yahya Alsayed, Mohsenah Jaber Alfaifi

**Affiliations:** 1 Faculty of Medical Laboratory Technology, Northern Borders University, Arar, Saudi Arabia; 2 Nursing Administration Department, Huraymala General Hospital, Third Health Cluster, Riyadh, Saudi Arabia; 3 Department of Medical Surgical Nursing, Faculty of Nursing, King Saud University, Riyadh, Saudi Arabia; 4 Faculty of Medicine, Taibah University, Madinah, Saudi Arabia; 5 Faculty of Medicine, Najran University, Najran, Saudi Arabia; 6 Faculty of Medicine, Ibn Sina College, Jeddah, Saudi Arabia; 7 Faculty of Medicine, Al-Rayan National Colleges, Madinah, Saudi Arabia; 8 Nursing Specialist, Faculty of Nursing, Umm Al-Qura University, Makkah, Saudi Arabia; 9 Jazan Specialist Hospital at Jazan Health Cluster, Jazan, Saudi Arabia

**Keywords:** Latent tuberculosis infection (LTBI), Healthcare workers (HCWs), Tuberculosis (TB) prevalence, Occupational health, Saudi Arabia, Systematic review

## Abstract

**Background:**

Tuberculosis (TB) remains a major global public health concern, and healthcare workers (HCWs) face an elevated risk of latent tuberculosis infection (LTBI) due to occupational exposure. Estimating prevalence among HCWs is therefore essential for informing infection control strategies. In Saudi Arabia, infection risk may be influenced by the multinational composition of the healthcare workforce and differing baseline TB exposure across countries of origin. Heterogeneous Bacillus Calmette–Guérin (BCG) vaccination histories may also introduce diagnostic bias in studies relying on the tuberculin skin test (TST). However, existing studies are predominantly single-center or regional, employ heterogeneous diagnostic methods, and report inconsistent prevalence estimates, precluding a national-level synthesis. Accordingly, we conducted a systematic review and meta-analysis to estimate LTBI prevalence among HCWs in Saudi Arabia and to examine factors associated with infection risk.

**Methods:**

We conducted a systematic review and meta-analysis in accordance with PRISMA 2020 guidelines. MEDLINE (via PubMed), Web of Science, Google Scholar, and the Elton B. Stephens Company (EBSCO) databases were searched up to March 2025 (final search conducted in March 2025), without applying a lower date limit, for observational studies reporting LTBI prevalence among adult HCWs in Saudi Arabia. LTBI was diagnosed using tuberculin skin tests or interferon-gamma release assays. Random-effects models were used to estimate pooled prevalence and to derive odds ratios from within-study subgroup comparisons. Pre-specified subgroup analyses explored potential sources of heterogeneity by nationality, occupation, sex, and age. Methodological quality was assessed using the Appraisal Tool for Cross-Sectional Studies (AXIS) and the Methodological Index for Non-Randomized Studies (MINORS).

**Results:**

Eleven studies comprising 11,249 HCWs were included. The pooled prevalence of LTBI was 24% (95% CI: 16–33%), with substantial heterogeneity (I^2^ = 99%). Sensitivity analysis excluding one high-prevalence study reduced the pooled estimate to 19%. Subgroup analyses showed that Saudi HCWs had significantly lower odds of LTBI compared with non-Saudi HCWs (OR = 0.31, 95% CI: 0.21–0.46). Nurses had higher odds of LTBI than technicians (OR = 1.40, 95% CI: 1.07–1.83), while no significant differences were observed between nurses and physicians or between male and female HCWs. LTBI prevalence varied across age categories, from 16% among HCWs younger than 30 years to 26% among those aged 50 years or older.

**Conclusion:**

LTBI remains a significant occupational health concern among HCWs in Saudi Arabia, with higher prevalence observed among non-Saudi staff and nurses. However, the available evidence does not allow these differences to be attributed to specific causes, as key factors such as country of origin, BCG status, and non-occupational exposure were not consistently reported. Strengthened TB control policies, including standardised screening protocols, fit-testing, and consistent protective measures, are needed to improve early detection and reduce transmission risk*.*

**Supplementary Information:**

The online version contains supplementary material available at 10.1186/s12889-026-27751-0.

## Introduction

Tuberculosis (TB) is a preventable yet persistent infectious disease caused by *Mycobacterium tuberculosis* and remains a major global public health concern [1, 2]. Although TB affects diverse populations, healthcare workers (HCWs) represent a particularly high-risk group because of frequent occupational exposure to infectious patients and aerosol-generating procedures [3].

TB occurs in two clinical forms: active TB, which is symptomatic and infectious, and latent tuberculosis infection (LTBI), in which individuals are infected but asymptomatic and non-contagious [4]. Although clinically silent, LTBI may reactivate over time, with reactivation risk increased in immunosuppressed individuals (e.g., HIV infection, corticosteroid or biologic therapy, malnutrition, end-stage renal disease) [5, 6]. In healthcare settings, repeated occupational exposure does not accelerate reactivation of existing LTBI; rather, it increases the risk of new infection or reinfection, either of which may subsequently progress to active disease. While individuals with LTBI are not infectious, undetected progression to active disease poses a risk to both HCWs and patients in healthcare settings [6].

Transmission of *M. tuberculosis* occurs through airborne particles generated by individuals with active pulmonary disease. In healthcare settings, occupational risk is shaped by several factors, including close and repeated contact with patients harbouring undiagnosed or recently diagnosed pulmonary TB; participation in aerosol-generating procedures such as bronchoscopy, sputum induction, intubation, suctioning, and non-invasive ventilation; performance of laboratory work involving viable mycobacterial cultures; and exposure to inadequately ventilated clinical and waiting areas. Risk is further modulated by departmental assignment (e.g., pulmonology, infectious diseases, intensive care, emergency departments), the duration and frequency of patient contact, and the timeliness of diagnosis and isolation of infectious cases [7]. Consequently, international guidelines recommend routine TB screening and preventive strategies for HCWs and other high-risk populations [8]. Screening for LTBI relies on either the tuberculin skin test (TST) or interferon-gamma release assays (IGRAs), which differ in sensitivity and specificity, particularly in Bacillus Calmette–Guérin (BCG)-vaccinated populations and multinational workforces with heterogeneous BCG vaccination policies and TB exposure backgrounds [9].

According to recent WHO reports, approximately one-quarter of the global population is estimated to be infected with *Mycobacterium tuberculosis* [2]. In Saudi Arabia, TB incidence is estimated at 8–10 cases per 100,000 population [2]. Nevertheless, LTBI prevalence estimates vary considerably, ranging from 9.8% in the general population to approximately 26–27% among HCWs [10]. A recent national systematic review reported an LTBI prevalence of 17.0%, exceeding the global average [10]. Global evidence suggests that HCWs in high-exposure settings may face a 10–20-fold higher risk of active TB compared with the general population, reflecting cumulative occupational exposure rather than community transmission alone [11–13].

Several studies have examined *Mycobacterium tuberculosis* infection among HCWs in Saudi Arabia [14–16]; however, most were limited to single institutions or regions, applied heterogeneous diagnostic approaches, and lacked national representativeness. As a result, no comprehensive national-level synthesis currently exists to estimate LTBI prevalence or to systematically identify demographic and occupational risk patterns among HCWs. International guidelines emphasise the importance of evidence-based LTBI screening and infection-control strategies for HCWs [17], underscoring the need for consolidated national data to inform policy and practice in Saudi Arabia.

Therefore, we conducted a systematic review and meta-analysis to estimate the prevalence of LTBI among HCWs in Saudi Arabia. Additionally, we aimed to examine variation in prevalence across demographic and occupational subgroups, assess the methodological quality of included studies, and generate evidence to support TB prevention and control strategies. To our knowledge, this study represents the first comprehensive synthesis of LTBI prevalence among HCWs in Saudi Arabia, incorporating formal quality appraisal.

## Methods

### Literature search strategy

This systematic review was conducted in accordance with the PRISMA 2020 guidelines [18]. In addition, key principles from the Meta-Analysis of Observational Studies in Epidemiology (MOOSE) guidelines were considered to inform the assessment of bias, heterogeneity, and cautious interpretation of findings derived from observational studies. The study protocol was prospectively registered in PROSPERO (ID: CRD420251033222).

We systematically searched MEDLINE (via PubMed), Web of Science, Google Scholar, and the Elton B. Stephens Company (EBSCO) databases from January 2000 to March 2025 (final search conducted in March 2025). Google Scholar was used as a supplementary source to identify studies not captured in structured databases; the first 200 results (sorted by relevance) were screened. Due to limited reproducibility, it was not considered a primary database. To minimise inclusion of non–peer-reviewed sources retrieved via Google Scholar, only full-text journal articles were included, and theses, conference abstracts, and unpublished reports were excluded. All search results were exported, merged in EndNote, and automatically deduplicated, followed by manual duplicate verification before screening in Rayyan. The search strategy combined Medical Subject Headings (MeSH) and keywords: (“epidemiology” OR “prevalence” OR “incidence”) AND (“tuberculosis” OR “TB” OR “Mycobacterium tuberculosis” OR “M. tuberculosis”) AND (“Saudi Arabia” OR “KSA” OR “Kingdom of Saudi Arabia”) AND (“healthcare worker” OR “HCW” OR “healthcare provider” OR “physician” OR “nurse”). Boolean operators were used to optimise sensitivity and specificity. The inclusion criteria followed the population, intervention, comparison, outcome, timing, and settings (PICTOS) framework, which guided both the search strategy and eligibility criteria: Population = adult HCWs; Intervention/Exposure = occupational risk of TB; Comparison = not applicable; Outcome = LTBI prevalence; Timing = studies with clearly defined data collection periods; Setting = healthcare facilities in Saudi Arabia.

### Eligibility criteria

We included studies reporting LTBI prevalence among adult HCWs, defined as clinical or nonclinical staff employed in healthcare settings. Students, interns, and trainees were excluded unless they were explicitly classified as HCWs in the original studies, to ensure consistency in occupational exposure definitions. Eligible studies were limited to observational designs, including cross-sectional, retrospective, or cohort studies. They were required to present original data published as full-text journal articles in English or Arabic. Cohort studies were treated as observational studies for this review.

We excluded non-original articles, such as editorials, letters, commentaries, and review articles, as well as those focusing on patient populations rather than HCWs, lacking relevant prevalence or incidence data, or addressing TB in non-occupational contexts. Conference abstracts, letters, theses, and unpublished reports were also excluded. In addition, studies deemed to have poor methodological quality (e.g., unclear data collection or small sample sizes) were recorded and considered during interpretation but were not used as exclusion criteria.

### Study selection and data extraction

Two reviewers (Y.N.A. and A.M.A.) independently screened the titles, abstracts, and full texts using Rayyan software. Data were extracted using a standardised form to capture study characteristics, participant demographics, diagnostic methods, and LTBI prevalence estimates. Disagreements at any stage were resolved through discussion, with consultation of a third reviewer (A.S.B.) when consensus could not be reached.

### Quality assessment

Methodological quality and risk of bias were evaluated using validated tools according to study design. Cross-sectional studies were appraised using the Appraisal Tool for Cross-Sectional Studies (AXIS) [19], which includes 20 items covering the study design, methodology, and reporting quality. Retrospective observational studies were assessed using the Methodological Index for Non-Randomized Studies (MINORS) [20], comprising eight core criteria (scored from 0 to 2). Two independent reviewers conducted the assessments, and discrepancies were resolved by consensus. Inter-rater agreement between reviewers was quantified prior to consensus using percent agreement, with disagreements resolved through discussion. Quality scores informed the interpretation but were not used as exclusion criteria. Studies scoring below 50% of the maximum possible AXIS or MINORS score were considered to have poor methodological quality, particularly when there was evidence of unclear sampling strategies, inadequate reporting, or missing diagnostic information. The certainty of the overall body of evidence was not formally assessed using the GRADE framework, as GRADE is primarily designed for intervention and guideline-focused reviews rather than prevalence studies.

### Meta-analysis and heterogeneity assessment

Pooled prevalence estimates and subgroup odds ratios were calculated using random-effects meta-analysis models to account for between-study variability. Statistical heterogeneity was assessed using the I^2^ statistic, with thresholds of 25%, 50%, and 75% indicating low, moderate, and high heterogeneity, respectively. Subgroup analyses by occupation, nationality, sex, and age were pre-specified in the protocol and conducted to explore potential sources of heterogeneity. Prediction intervals were considered but ultimately not reported, as the high heterogeneity and limited number of studies within each subgroup reduced their interpretability. Although diagnostic methods varied across studies (TST vs. IGRA), diagnostic modality was not analysed as a separate subgroup because only two studies provided extractable IGRA-only prevalence estimates, and several studies applied mixed or confirmatory testing approaches, limiting comparability. A *p*-value < 0.05 was considered statistically significant. To further explore potential sources of heterogeneity, univariate meta-regression analyses were performed using study-level covariates, including publication year, diagnostic method, and proportion of non-Saudi participants. Meta-regression analyses were conducted using random-effects models in R (version 4.5.1; R Foundation for Statistical Computing, Vienna, Austria).

### Publication bias

Publication bias was assessed using funnel plot symmetry and Egger’s regression test. All statistical analyses were conducted using STATA (StataCorp, College Station, TX, USA) software.

## Results

### Study selection

**Fig. 1:**
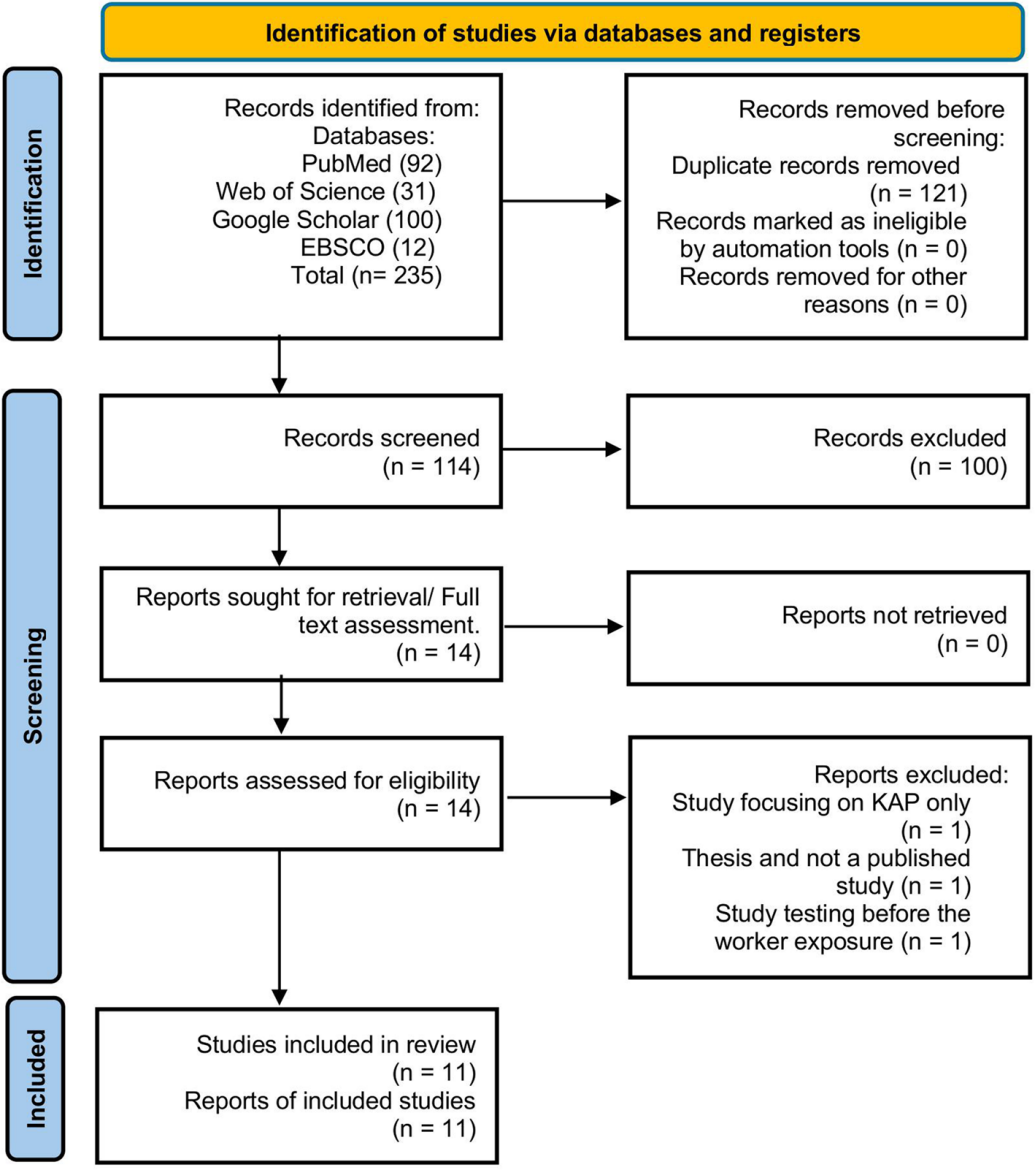
PRISMA 2020 flow diagram illustrating the study selection process for the systematic review and meta-analysis of latent tuberculosis infection (LTBI) prevalence among healthcare workers (HCWs) in Saudi Arabia

A total of 235 studies were identified through database searches (PubMed/Medline, *n* = 92; Web of Science, *n* = 31; Google Scholar, *n* = 100; and EBSCO, *n* = 12). After the removal of 121 duplicates, 114 studies were retained for screening. Of these, 100 were excluded based on titles and abstracts, and the remaining 14 full-text articles were assessed for eligibility. Among these selected articles, three were excluded (one study focused only on knowledge, attitudes, and practices (KAP); one was a thesis and not a peer-reviewed publication; and one assessed LTBI prior to occupational exposure). Finally, 11 studies were included in the systematic review and meta-analysis (Fig. 1).

### Study characteristics and quality

The included studies were published between 2003 and 2024 and enrolled a combined total of 11,249 HCWs from healthcare facilities across Saudi Arabia. Most studies used cross-sectional designs, and the diagnostic methods for LTBI varied across studies: eight studies used TST as the primary diagnostic method, whereas the remaining three studies used IGRA. Of the latter, only two studies provided extractable IGRA-only prevalence estimates; the remaining study applied both IGRA and TST in a manner that did not allow a diagnostic-specific prevalence estimate to be consistently extracted. Most studies were conducted in tertiary care hospitals, with some covering high-risk departments such as emergency, internal medicine, intensive care units, and infectious disease units. The study characteristics are summarised in Table 1. Notably, the study by Assiri et al. [26] was a retrospective observational study based on occupational health record review, reporting TST conversion as part of broader infection surveillance. Assessment of study quality using the AXIS and MINORS tools rated most studies as fair to good in quality.

**Table 1:** Characteristics of included studies. All prevalence values correspond to latent tuberculosis infection (LTBI), not active TB. Methodological quality was assessed using the Appraisal Tool for Cross-Sectional Studies (AXIS, maximum score 20) for cross-sectional designs and the Methodological Index for Non-Randomized Studies (MINORS, maximum score 16) for retrospective designs

Study	Nationality, *n* (%)	Study design	Population and Site	LTBI Diagnostic Method	Methodological quality	LTBI Prevalence%
Almugti, 2022 [21]	Saudi 63%, non-Saudi 37%	Cross-sectional study	HCWs across Saudi Arabia (no single recruitment site specified)	TST + IGRA + X-ray (screening)	AXIS: 17/20	19%
Alshahrani, 2023 [14]	Saudi 9.1%, non-Saudi 90.9%	Cross-sectional study	King Abdullah Hospital, Bisha; HCWs	TST	AXIS: 19/20	11.8%
Nabil, 2015 [22]	Saudi 11.5%, non-Saudi 88.5%	cross-sectional study	Muhayil National Hospital; HCWs	X-ray + TST	AXIS: 17/20	22.5%
Bukhary, 2018 [23]	Saudi 42.5%, non-Saudi 57.5%	Cross-sectional study	Multiple non-tertiary care hospitals, Madina; HCWs across professional roles	IGRA (QFT-GIT) + TST	AXIS: 19/20	10.8%
Almohaya, 2020 [15]	Saudi 24.8%, non-Saudi 74.2%	Cross-sectional and case–control study	Tertiary academic hospital, Riyadh; clinical and non-clinical staff across work areas	QFT-plus (IGRA only)	AXIS: 18/20	24.2%
Aldhawyan, 2024 [24]	Saudi 12.8%, non-Saudi 87.2%	Cross-sectional study	Tertiary academic hospital, Riyadh; nursing staff in critical areas	TST	AXIS: 18/20	34.5%
Balkhy, 2013 [16]	Saudi 16.1%, non-Saudi 83.9%	Retrospective study	King Abdulaziz Medical City, Riyadh; HCWs	TST + X-ray	MINORS: 11/16	21.1%
Al Hajoj, 2016 [25]	Not reported	Cross-sectional study	King Faisal Specialist Hospital and Research Centre, Riyadh; HCWs	TST	AXIS: 19/20	31.5%
Assiri, 2013 [26]	Not reported	Retrospective observational study (occupational health record review)	Secondary care hospital, Najran; HCWs (occupational health record review)	TST	MINORS: 10/16	4.2%
Koshak & Tawfeeq, 2003 [27]	Saudi 21%, non-Saudi 79%	Cross-sectional observational study	King Abdulaziz University Hospital health clinics, Jeddah; HCWs	TST	AXIS: 17/20	78.9%
Abbas, 2010 [28]	Saudi 47.38%, non-Saudi 52.62%	Cross-Sectional Study Design	Major tertiary care hospitals, Riyadh; HCWs	TST	AXIS: 17/20	11%

### Overall prevalence of latent tuberculosis infection (LTBI)

**Fig. 2:**
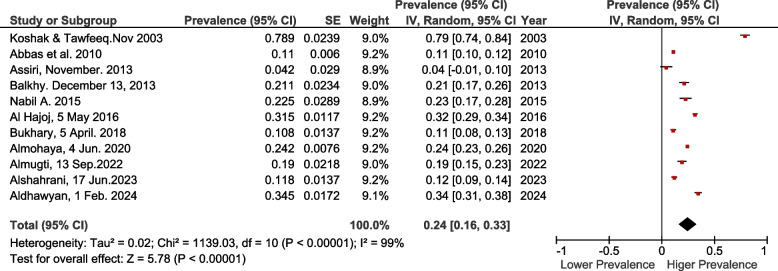
Forest plot of pooled prevalence of latent tuberculosis infection (LTBI) among healthcare workers (HCWs) in Saudi Arabia (pooled prevalence = 24.0%; 95% CI: 16–33%; *p* < 0.0001; I^2^ = 99%)

Eleven studies reporting the prevalence of LTBI were included in the pooled analysis. The overall pooled prevalence of LTBI among HCWs in Saudi Arabia was 24.0% (95% CI: 16–33%; *p* < 0.0001), with substantial heterogeneity across the studies (I^2^ = 99%). This high variability reflects differences in study settings, diagnostic methods, and population characteristics. These findings underscore the significant burden of latent TB infection in the Saudi healthcare workforce (Fig. 2).

### Sensitivity analysis

Sensitivity analysis excluding the study with the highest reported prevalence (78.9%; [27]) reduced the pooled LTBI prevalence from 24 to 19% and lowered heterogeneity from 99 to 82%. Although heterogeneity remained high, the direction and significance of the pooled estimate were unchanged, indicating that the overall findings were not driven by a single outlier study.

### Prevalence of latent tuberculosis infection (LTBI) by healthcare facility level

**Fig. 3:**
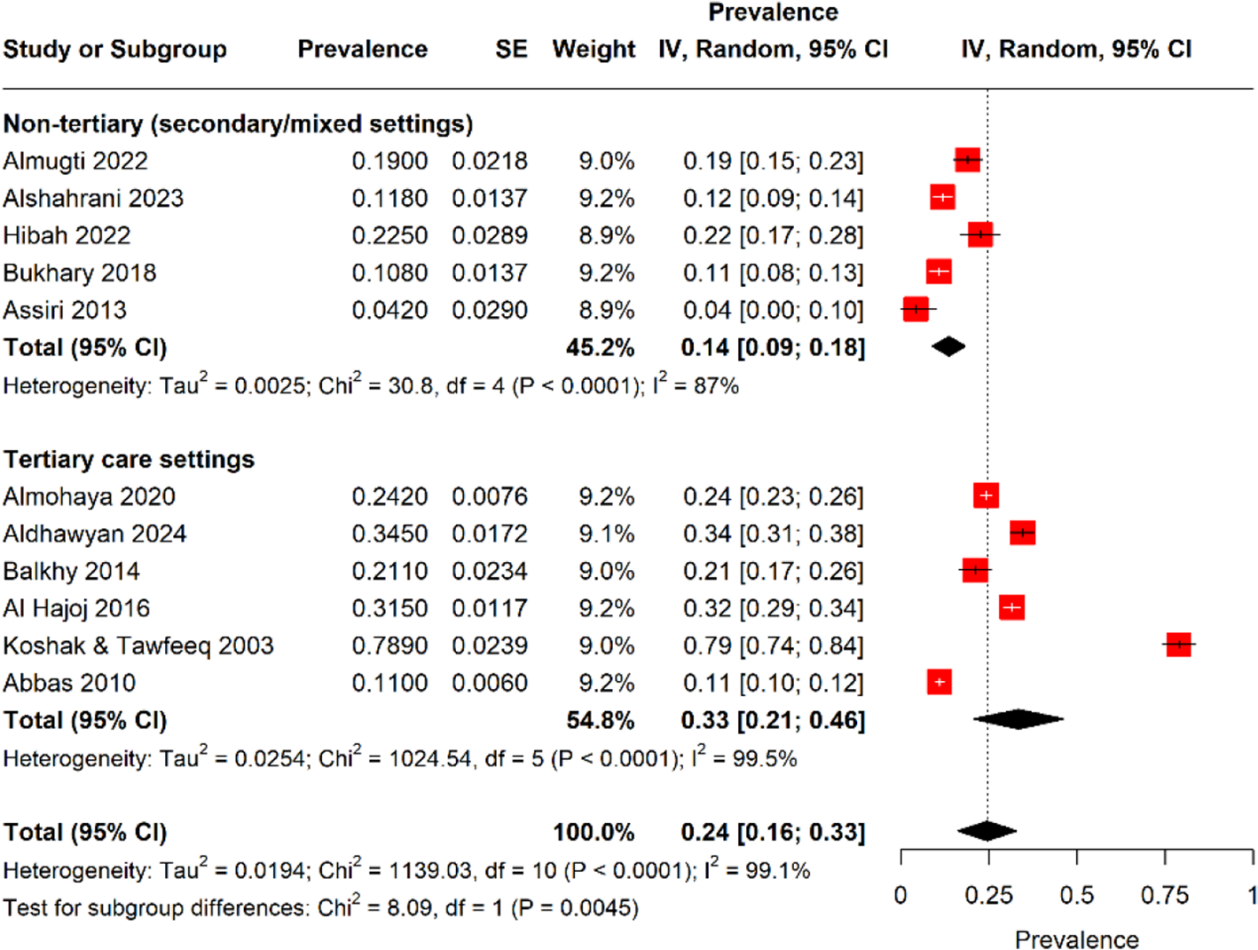
Forest plot of pooled latent tuberculosis infection (LTBI) prevalence among healthcare workers (HCWs) stratified by healthcare facility level. The pooled prevalence was higher in tertiary care settings (33.5%, 95% CI: 14.6–52.4%) compared with non-tertiary settings (13.6%, 95% CI: 7.6–19.6%), with a statistically significant subgroup difference (*p* = 0.0497)

Subgroup analysis by healthcare facility level demonstrated a higher pooled prevalence of LTBI among healthcare workers (HCWs) in tertiary care settings compared with non-tertiary settings. The pooled prevalence in tertiary hospitals was 33.5% (95% CI: 14.6–52.4%), whereas in non-tertiary settings it was 13.6% (95% CI: 7.6–19.6%). The difference between subgroups was statistically significant (Q = 3.85, *p* = 0.0497). Substantial heterogeneity was observed within both subgroups (I^2^ = 98% for tertiary and I^2^ = 87% for non-tertiary settings), indicating considerable variability across studies (Fig. 3).

### Prevalence of latent tuberculosis infection (LTBI) by nationality

**Fig. 4:**
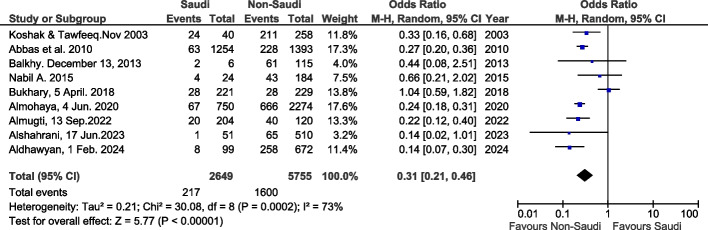
Forest plot comparing latent tuberculosis infection (LTBI) prevalence between Saudi and non-Saudi healthcare workers (HCWs) (pooled OR: 0.31; 95% CI: 0.21–0.46; *p* < 0.0001; I^2^ = 73%)

Subgroup analysis was conducted to compare the prevalence of LTBI between Saudi and non-Saudi HCWs. Data from nine studies revealed that non-Saudi workers had significantly higher odds of LTBI than Saudi nationals. The pooled odds ratio was 0.31 (95% CI: 0.21–0.46; *p* < 0.0001), indicating that non-Saudi HCWs had 3.2-fold higher odds of LTBI than Saudi HCWs. Substantial heterogeneity was observed across the studies (I^2^ = 73%); the sources of this heterogeneity could not be empirically attributed to specific factors within this analysis (see Discussion) (Fig. 4).

### Prevalence of latent tuberculosis infection (LTBI) by occupation: physicians vs. nurses

To evaluate the occupational risk of LTBI, we compared the prevalence between physicians and nurses in six studies. The pooled odds ratio was 0.83 (95% CI: 0.48–1.44; *p* = 0.52), indicating no significant difference between the two groups. Substantial heterogeneity was observed (I^2^ = 81%; *p* < 0.0001). While some studies reported a higher prevalence among physicians (Supplementary Figure S1).

### Prevalence of latent tuberculosis infection LTBI by occupation: nurses vs. technicians

**Fig. 5:**
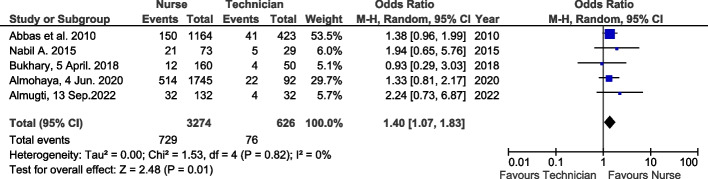
Forest plot comparing latent tuberculosis infection (LTBI) prevalence between nurses and technicians (pooled OR: 1.40; 95% CI: 1.07–1.83; *p* < 0.01; I^2^ = 0%)

Five studies assessed the prevalence of LTBI in nurses and technicians. The pooled odds ratio was 1.40 (95% CI: 1.07–1.83; *p* < 0.01), indicating a significantly higher prevalence among nurses than among technicians. Heterogeneity was negligible (I^2^ = 0%, *p* = 0.82) (Fig. 5).

### Prevalence of latent tuberculosis infection LTBI by occupation: physicians* vs.* technicians

Five studies compared the prevalence between physicians and technicians. The pooled odds ratio was 1.23 (95% CI: 0.70–2.18; *p* = 0.47), indicating no significant difference between the two groups. Moderate heterogeneity was observed (I^2^ = 56%, *p* < 0.06). These results suggest that, while physicians may experience earlier or more direct exposure to TB, technicians working in diagnostic or laboratory settings also face occupational risks (Supplementary Figure S2).

### Prevalence of latent tuberculosis infection LTBI by sex

Nine studies reported that the prevalence of LTBI disaggregated by sex. The pooled odds ratio for male versus female HCWs was 1.01 (95% CI: 0.75–1.38; *p* = 0.93), indicating no significant association between sex and LTBI status. Substantial heterogeneity was observed (I^2^ = 81%). These findings suggest that sex is not a consistent predictor of LTBI risk in this population (Supplementary Figure S3).

### Prevalence of latent tuberculosis infection LTBI by age group

Subgroup analysis was conducted to evaluate the prevalence of LTBI across different age categories. The pooled prevalence among HCWs aged < 30 years was 16% (95% CI: 8%–23%, *p* < 0.0001; I^2^ = 96%), based on six studies. In the 30–49-year age group, the pooled prevalence was 18% (95% CI: 14%–22%, *p* < 0.0001; I^2^ = 89%). The highest prevalence was observed in the ≥ 50-year age group, with a pooled estimate of 26% (95% CI: 16%–36%, *p* < 0.0001; I^2^ = 81%). The overall pooled prevalence across all age strata was 19% (95% CI: 14%–24%, *p* < 0.0001; I^2^ = 97%), with significant heterogeneity between subgroups (χ^2^ = 281.04, df = 2, *p* < 0.0001). These findings suggest a possible increase in LTBI prevalence with age; however, no formal test for trend was performed (Supplementary Figure S4).

### Meta-regression analyses

**Fig. 6:**
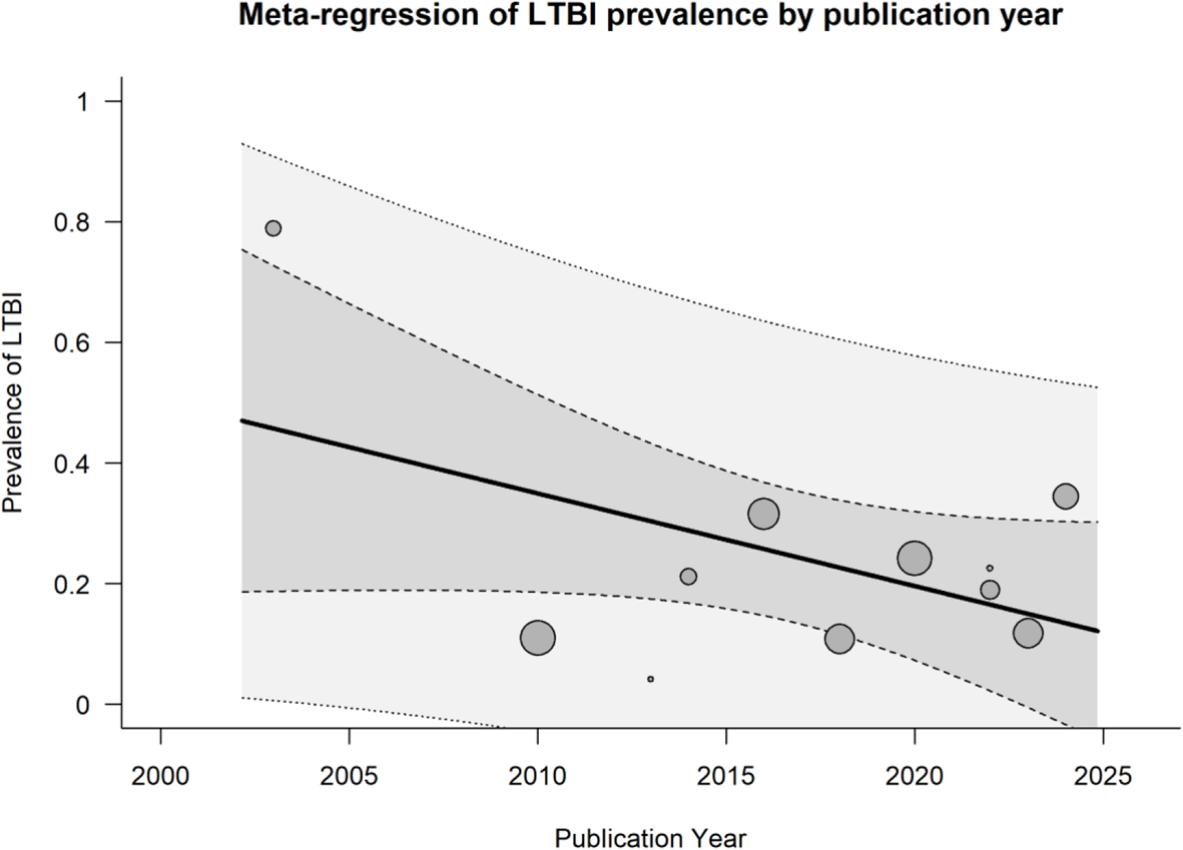
Meta-regression of LTBI prevalence by publication year. Bubble size reflects study weight. The solid line represents the fitted regression, with shaded 95% confidence intervals and dotted prediction intervals

Meta-regression analyses were conducted to explore potential sources of between-study heterogeneity using three predefined study-level covariates: publication year, diagnostic method, and proportion of non-Saudi participants. None of the examined moderators significantly explained the observed heterogeneity. Publication year showed a non-significant inverse association with LTBI prevalence (*p* = 0.091), accounting for 15.6% of the variance. Diagnostic method (TST-only vs. mixed/IGRA) was not significantly associated with LTBI prevalence (*p* = 0.466; R^2^ = 0.00%). Similarly, the proportion of non-Saudi participants showed a non-significant positive association (*p* = 0.188), explaining 6.85% of the variance. Residual heterogeneity remained high (I^2^ > 99%), indicating that the included study-level variables did not adequately account for the variability in prevalence estimates. The relationship between LTBI prevalence and publication year is illustrated in Fig. 6.

### Publication bias

The risk of publication bias was assessed using funnel plots and Egger’s test. Visual inspection suggested funnel plot asymmetry (Supplementary Figure S5), whereas Egger’s test showed no significant publication bias (*p* = 0.64), indicating that the observed asymmetry may not be due to small study effects or publication bias alone. Therefore, the evidence of publication bias in this meta-analysis remains inconclusive.

### Study quality

Quality assessment indicated variability in the methodological rigour of the included studies. Nine cross-sectional studies were assessed using the AXIS tool, yielding total scores between 17 and 19 out of 20, signifying overall high methodological quality. Most studies exhibited clearly articulated objectives, appropriate study designs, and thoroughly documented data. Nonetheless, common limitations included insufficient justification for sample size, inadequate reporting on non-responders, and missing data.

Two retrospective non-randomised studies were evaluated using the MINORS tool, achieving total scores of 11 and 10 out of a maximum score of 16. Although both studies explicitly articulated their objectives and established appropriate endpoints and follow-up, they were deficient in prospective sample size calculation, blinded outcome assessment, and comparative group data, indicating moderate methodological quality. A comprehensive summary of the AXIS and MINORS scores is presented in Table 2 Supplementary.

## Discussion

This systematic review and meta-analysis synthesised evidence from 11 studies conducted across diverse healthcare settings in Saudi Arabia and demonstrates that LTBI remains a substantial occupational health concern among HCWs. Although Saudi Arabia is classified as a low TB-burden country, the findings indicate that LTBI prevalence among HCWs is substantial, underscoring the ongoing relevance of occupational exposure, workforce composition, and screening practices in shaping infection risk. The estimated LTBI prevalence among HCWs in Saudi Arabia is lower than that reported in high TB-burden countries such as Iran (47.8%) [29, 30] and Egypt (44.9%) [31, 32]. However, it remains substantially higher than that reported in low-burden Gulf countries, including the United Arab Emirates (1%) [33] and Qatar (6.9%) [34]. Direct comparisons across countries should be interpreted cautiously, given differences in diagnostic methods, study design, and workforce composition. As discussed below, the country of origin of non-Saudi HCWs was not reported in the included studies, which limits our ability to compare cross-country patterns directly.

The inability to conduct a diagnostic-specific subgroup analysis represents a major limitation of this review. Although only two diagnostic modalities are in standard use, TST and IGRA, included studies varied in which modality they applied, in TST induration cutoffs, in whether BCG status was adjusted for, and in whether TST and IGRA were used sequentially or in combination. Most included studies relied on TST alone, while only a small subset employed IGRA, sometimes in combination with TST.

Importantly, the majority of included studies relied on TST, which may yield false-positive results in BCG-vaccinated individuals, and none of the included studies consistently adjusted for BCG vaccination status. A more fundamental limitation also applies to both TST and IGRA: these are immune-memory–based assays that detect a host immune response to *M. tuberculosis* antigens, not the presence of viable bacilli. A positive result indicates immunologic sensitisation and does not by itself distinguish among persistent latent infection, past resolved infection, or prior treated infection. As a result, "LTBI prevalence" estimates derived from these tools, including those synthesised in this review, should be interpreted as prevalence of immunological evidence of prior *M. tuberculosis* exposure rather than as prevalence of currently established infection. A similar prevalence pattern was observed in the study using IGRA-only testing, suggesting that the observed nationality difference may not be solely explained by test-related bias; however, the diagnostic and interpretive limitations described above mean that nationality-based comparisons should be interpreted with caution.

Beyond estimating prevalence, the findings have important operational implications for healthcare systems. In clinical environments, even a moderate LTBI burden increases the need for reliable screening, timely referral, and preventive therapy programs to ensure continuity of workforce safety. Undetected LTBI in high-exposure departments may strain occupational health resources and increase the workload associated with contact tracing and surveillance activities. These considerations underscore the need for coordinated institutional policies that prioritise routine monitoring and targeted prevention and early intervention strategies for HCWs.

Several non-mutually-exclusive explanations could in principle contribute to the higher LTBI prevalence observed among non-Saudi HCWs. These include exposure occurring before migration to Saudi Arabia, non-occupational exposure within Saudi Arabia, differential occupational exposure across departments, and diagnostic misclassification from TST cross-reactivity in BCG-vaccinated individuals. However, BCG status, country of origin, housing conditions, and departmental distribution stratified by nationality were not consistently reported across the included studies and therefore cannot be used to explain the nationality difference. We attempted to further stratify non-Saudi healthcare workers by country or region of origin, but the included studies reported nationality only as Saudi versus non-Saudi, without additional geographic detail. The higher LTBI prevalence among non-Saudi HCWs should therefore be reported as an observation rather than explained, and policy implications drawn from this association should be correspondingly cautious.

Between-study heterogeneity was exceptionally high, indicating that the included studies were unlikely to be estimating a single underlying prevalence. Variability in diagnostic methods, healthcare settings, workforce composition, and screening protocols all contributed to this heterogeneity. Subgroup analysis by healthcare facility level was feasible in this study and demonstrated a higher prevalence of LTBI among healthcare workers in tertiary care settings compared with non-tertiary settings. This finding could be consistent with greater occupational exposure in tertiary hospitals, although the included studies did not provide study-level data on patient volume, case mix, aerosol-generating procedure exposure, or infection-control practices that would allow this hypothesis to be tested directly. However, this finding should be interpreted with caution given the substantial heterogeneity observed across studies and the variability in reporting of healthcare facility characteristics. Furthermore, classification of healthcare settings was based on available study descriptions and may not fully capture differences in exposure intensity or infection control practices. Although sensitivity analyses demonstrated that individual outlier studies influenced the magnitude of pooled estimates, the overall direction of the pooled estimates remained stable. Subgroup analyses by nationality, healthcare facility level, occupation, and age accounted for some of the observed variability, although these analyses were exploratory and cannot establish causal determinants. Meta-regression analyses using publication year, diagnostic method, and proportion of non-Saudi participants did not identify any significant source of heterogeneity. We therefore do not attribute the observed heterogeneity to any specific factor. Several other potentially relevant moderators, including BCG vaccination status, country of origin, departmental assignment, and TST cutoff conventions were not consistently reported across studies (see preceding paragraph) and could not be evaluated. The pooled prevalence should accordingly be interpreted as a descriptive summary of a heterogeneous body of evidence rather than a precise estimate of a single underlying parameter.

The assessment of publication bias using funnel plot asymmetry and Egger’s test in this review suggests inconclusive evidence of publication bias. Funnel plot asymmetry does not necessarily indicate publication bias, particularly in meta-analyses with a small number of studies and substantial between-study heterogeneity. In such contexts, visual asymmetry may arise from true clinical and methodological differences rather than selective publication and should therefore be interpreted with caution. In addition, Egger’s regression test has limited statistical power when fewer than ten to fifteen studies are included.

LTBI screening practices in Saudi Arabia remain variable across healthcare institutions in the absence of a unified national policy governing routine HCW testing. Most healthcare institutions rely on TST for pre-employment screening, with IGRAs used selectively, particularly among BCG-vaccinated staff or when confirmation is required. Periodic or repeated screening is applied inconsistently and is often restricted to high-risk departments or post-exposure scenarios. This institutional variability likely contributes to differences in LTBI detection, follow-up practices, and reporting across studies.

Occupational subgroup analyses indicated a higher LTBI prevalence among nurses than among technicians, which is consistent with their more frequent and prolonged patient contact, particularly in clinical wards where TB exposure risk is elevated. However, occupational risk appears to be influenced more strongly by departmental assignment than by job title alone. HCWs working in pulmonary and infectious disease units face substantially higher exposure risk than those working in non-clinical or diagnostic units, a pattern that has been reported in international studies [8, 29, 35]. The absence of an association between sex and LTBI, despite higher prevalence among nurses, reflects the specific workforce composition in Saudi Arabia, where nursing includes a substantial proportion of male staff. This distinction suggests that observed differences are driven by occupational exposure rather than sex-based biological susceptibility. These occupational subgroup comparisons were conducted as exploratory analyses and should be interpreted with caution. As multiple pairwise comparisons were performed without a single unified reference model, there is an increased risk of type I error. However, given the limited number of studies and the absence of individual-level data, more complex analytical approaches, such as regression-based or network meta-analytic models, were not feasible. Nonetheless, the consistency of directional findings across studies lends support to their interpretive value despite these limitations.

To our knowledge, this review represents the first comprehensive synthesis of LTBI prevalence among HCWs in Saudi Arabia incorporating formal quality appraisal using the AXIS and MINORS tools. While methodological quality was generally fair to good, common limitations included inadequate sample size justification, incomplete reporting of non-responders, and variability in diagnostic protocols. These factors likely contributed to heterogeneity and should be considered when interpreting pooled estimates. Data on active TB among HCWs were extremely limited, precluding meaningful assessment of progression from latent to active disease. Consequently, the findings primarily reflect LTBI prevalence rather than TB incidence. Finally, restriction to studies published in English and Arabic may have introduced language bias. Although English and Arabic represent the primary languages of scientific publication in Saudi Arabia, some relevant evidence published in other languages may have been missed. Additionally, the use of Google Scholar as a supplementary search source introduces limitations related to reproducibility and precise record tracking.

## Conclusion

LTBI represents a substantial occupational health concern among HCWs in Saudi Arabia. Reported prevalence varied widely across the included studies, and extreme between-study heterogeneity combined with inconsistent reporting of key variables (described in the Discussion) precludes a single precise national estimate.

The available data do not support nationality-based screening recommendations. The findings support cautious discussion of standardised screening strategies for HCWs, while stronger prospective evidence is needed before making specific policy recommendations. Consistent with existing WHO guidance on LTBI management [17], future occupational TB studies should also routinely report departmental, demographic, and exposure data. Determining the optimal screening strategy will require stronger prospective evidence than this synthesis provides, and any policy implementation should be informed by national stakeholders drawing on broader evidence than that synthesised here.

Future research should prioritise longitudinal cohort studies that report country of origin, BCG status, departmental assignment, and serial testing data, since these are the variables most likely to clarify the determinants of LTBI risk in this workforce. Adoption of standardised diagnostic criteria and uniform reporting across healthcare institutions would also improve comparability of findings and reduce variability in future meta-analyses.

Overall, this review provides the first comprehensive national synthesis of LTBI prevalence among HCWs in Saudi Arabia and offers a foundation for future research and policy discussion in this area.

## Supplementary Information

Supplementary Material 1: Supplementary Figure S1. Forest plot comparing latent tuberculosis infection (LTBI) prevalence between physicians and nurses (pooled OR: 0.83; 95% CI: 0.48–1.44; *p* = 0.52; I² = 81%). Supplementary Figure S2. Forest plot comparing latent tuberculosis infection (LTBI) prevalence between physicians and technicians (pooled OR: 1.23; 95% CI: 0.70–2.18; *p* = 0.47; I² = 56%). Supplementary Figure S3. Forest plot comparing latent tuberculosis infection (LTBI) prevalence between male and female healthcare workers (HCWs) (pooled OR: 1.01; 95% CI: 0.75–1.38; *p* = 0.93; I2 = 81%). Supplementary Figure S4. Forest plot showing pooled latent tuberculosis infection (LTBI) prevalence among healthcare workers (HCWs) by age group. The pooled prevalence was 16% (95% CI: 8%–23%) for those aged < 30 years, 18% (95% CI: 14%–22%) for those aged 30–49 years, and 26% (95% CI: 16%–36%) for those aged ≥ 50 years. Overall heterogeneity was high (I2 = 97%, *p* < 0.0001). Supplementary Figure S5. Funnel plot assessing publication bias in studies on latent tuberculosis infection (LTBI) prevalence among healthcare workers (HCWs).

## Data Availability

All data generated or analysed in this study are included in the published article [and its supplementary information files].

## Abbreviations

TB: Tuberculosis
MTB: *Mycobacterium tuberculosis*
LTBI: Latent Tuberculosis Infection
MDR-TB: Multidrug-Resistant Tuberculosis
HCWs: Healthcare Workers
TST: Tuberculin Skin Test
IGRA: Interferon-Gamma Release Assay
QFT-GIT: QuantiFERON-TB Gold In-Tube test
QFT-Plus: QuantiFERON-TB Gold Plus test
AFB: Acid-Fast Bacilli
WHO: World Health Organization
HIV: Human Immunodeficiency Virus
DM: Diabetes Mellitus
AXIS: Appraisal Tool for Cross-Sectional Studies
MINORS: Methodological Index for Non-Randomized Studies
PRISMA: Preferred Reporting Items for Systematic Reviews and Meta-Analyses
PROSPERO: International Prospective Register of Systematic Reviews
OR: Odds Ratio
CI: Confidence Interval
