# Supplementary material for: Prevalence of latent tuberculosis infection among healthcare workers in Saudi Arabia: a systematic review and meta-analysis
Source: BMC Public Health. 2026 Jun 1;26:2339. doi: 10.1186/s12889-026-27751-0 (PMC13466511; doi:10.1186/s12889-026-27751-0)
Supplement: Supplementary file 1 [file 12889_2026_27751_MOESM1_ESM.zip › MINORS Quality Assessment Supplementary Data.docx]

|  |  | **D1** | **D2** | **D3** | **D4** | **D5** | **D6** | **D7** | **D8** | **Overall** |
| --- | --- | --- | --- | --- | --- | --- | --- | --- | --- | --- |
| **1** | **Balkhy. 2013** | 2 | 2 | 1 | 2 | 1 | 2 | 1 | 0 | 11 |
| **2** | **Assiri et al. 2013** | 1 | 1 | 1 | 1 | 0 | 1 | 0 | 0 | 5 |

Judgment

0 = Not reported

1 = Reported but inadequate

2 = Reported and adequate

D1: 1 A clearly stated aim

D2: 2 Inclusion of consecutive patients

D3: 3 Prospective collections of data

D4: 4 Endpoints appropriate to the aim of the study

D5: 5 Unbiased assessments of the study endpoint

D6: 6 Follow-up period appropriate to the aim of the study

D7: 7 Loss to follow-up less than 5%

D8: 8 Prospective calculations of the study size
