# Supplementary material for: Prevalence of latent tuberculosis infection among healthcare workers in Saudi Arabia: a systematic review and meta-analysis
Source: BMC Public Health. 2026 Jun 1;26:2339. doi: 10.1186/s12889-026-27751-0 (PMC13466511; doi:10.1186/s12889-026-27751-0)
Supplement: Supplementary file 1 [file 12889_2026_27751_MOESM1_ESM.zip › Quality Assessment Supplementary Data.docx]

|  |  | **D1** | **D2** | **D3** | **D4** | **D5** | **D6** | **D7** | **D8** | **D9** | **D10** | **D11** | **D12** | **D13** | **D14** | **D15** | **D16** | **D17** | **D18** | **D19** | **D20** | **D21** | **D22** | **Overall** |
| --- | --- | --- | --- | --- | --- | --- | --- | --- | --- | --- | --- | --- | --- | --- | --- | --- | --- | --- | --- | --- | --- | --- | --- | --- |
| **1** | **Al Hajoj, 2016** | 1 | 1 | 0 | 1 | 1 | 1 | 0 | 1 | 1 | 1 | 1 | 1 | 1 | 1 | 0 | 1 | 1 | 1 | 1 | 1 | 1 | 1 | 19 |
| **2** | **Hibah, 2015** | 1 | 1 | 0 | 1 | 1 | 1 | 0 | 1 | 1 | 1 | 1 | 1 | 1 | 1 | 0 | 1 | 1 | 1 | 0 | 1 | 1 | 0 | 17 |
| **3** | **Bukhary, 2018**  **Study** | 1 | 1 | 0 | 1 | 1 | 1 | 0 | 1 | 1 | 1 | 1 | 1 | 1 | 1 | 0 | 1 | 1 | 1 | 1 | 1 | 1 | 1 | 19 |
| **4** | **Almugti, 2022** | 1 | 1 | 0 | 1 | 1 | 0 | 0 | 1 | 1 | 1 | 1 | 1 | 0 | 0 | 1 | 1 | 1 | 1 | 1 | 1 | 1 | 1 | 17 |
| **5** | **Alshahrani, 2023** | 1 | 1 | 0 | 1 | 1 | 1 | 0 | 1 | 1 | 1 | 1 | 1 | 1 | 0 | 1 | 1 | 1 | 1 | 1 | 1 | 1 | 1 | 19 |
| **6** | **Aldhawyan, 2024** | 1 | 1 | 0 | 1 | 1 | 1 | 0 | 1 | 1 | 1 | 1 | 1 | 1 | 0 | 1 | 1 | 1 | 1 | 0 | 1 | 1 | 1 | 18 |
| **7** | **koshak & Tawfeeq, 2003** | 1 | 1 | 0 | 1 | 1 | 1 | 0 | 1 | 1 | 0 | 1 | 1 | 1 | 0 | 1 | 1 | 1 | 1 | 0 | 1 | 1 | 1 | 17 |
| **8** | **Almohaya, 2020** | 1 | 1 | 0 | 1 | 1 | 1 | 0 | 1 | 1 | 1 | 1 | 1 | 0 | 0 | 1 | 1 | 1 | 1 | 1 | 1 | 1 | 1 | 18 |
| **9** | **Abbas, 2010** | 1 | 1 | 0 | 1 | 1 | 1 | 0 | 1 | 1 | 1 | 1 | 1 | 0 | 0 | 1 | 1 | 1 | 1 | 1 | 0 | 1 | 1 | 17 |

D1: 1 Were the aims/objectives of the study clear?

Judgment

1= Yes

0= No

D2: 2 Was the study design appropriate for the stated aim (s)?

D3: 3 Was the sample size justified?

D4: 4 Was the target/reference population clearly defined?

D5: 5 Was the sample frame taken from an appropriate population base so that it closely

represented the target/reference population under investigation?

D6: 6 Was the selection process likely to select subjects/participants that were representative

of the target/reference population under investigation?

D7: 7 Were measures undertaken to address and categorise non-responders?

D8: 8 Were the risk factor and outcome variables measured appropriate to the aims of the

study?

D9: 9 Were the risk factor and outcome variables measured correctly using instruments/

measurements that had been trialled, piloted or published previously?

D10: 10 Is it clear what was used to determined statistical significance and/or precision

estimates?

D11: 11 Were the methods (including statistical methods) sufficiently described to enable them

to be repeated?

D12: 12 Were the basic data adequately described?

D13: 13 Does the response rate raise concerns about non-response bias?

D14: 14 Participant characteristics described

D15: 15 Missing data reported

D16: 16 Were the results internally consistent?

D17: 17 Were the results for the analyses described in the methods, presented?

D18: 18 Were the authors’ discussions and conclusions justified by the results?

D19: 19 Were the limitations of the study discussed?

D20: 20 Were there any funding sources or conflicts of interest that may affect the authors’

interpretation of the results?

D21: 21 Was ethical approval or consent of participants attained?

D22: 22 Other strengths/weaknesses
