# Supplementary material for: Prevalence of latent tuberculosis infection among healthcare workers in Saudi Arabia: a systematic review and meta-analysis
Source: BMC Public Health. 2026 Jun 1;26:2339. doi: 10.1186/s12889-026-27751-0 (PMC13466511; doi:10.1186/s12889-026-27751-0)
Supplement: Supplementary file 1 [file 12889_2026_27751_MOESM1_ESM.zip › Search Strategy Table.docx]

| Database | Full Search String / Strategy | Notes |
| --- | --- | --- |
| PubMed / MEDLINE | ("tuberculosis"[MeSH Terms] OR "Mycobacterium tuberculosis"[MeSH Terms] OR "tuberculosis"[Title/Abstract] OR "TB"[Title/Abstract]) AND ("prevalence"[Title/Abstract] OR "epidemiology"[Title/Abstract] OR "incidence"[Title/Abstract]) AND ("healthcare worker"[Title/Abstract] OR "healthcare workers"[Title/Abstract] OR "HCW"[Title/Abstract] OR "nurse"[Title/Abstract] OR "physician"[Title/Abstract]) AND ("Saudi Arabia"[Title/Abstract] OR "Kingdom of Saudi Arabia"[Title/Abstract] OR "KSA"[Title/Abstract]) | Filters: No date restrictions; Humans; English & Arabic |
| Web of Science (WoS) | TS = ((tuberculosis OR "Mycobacterium tuberculosis" OR TB) AND (prevalence OR epidemiology OR incidence) AND ("healthcare worker" OR HCW OR nurse OR physician) AND ("Saudi Arabia" OR "Kingdom of Saudi Arabia" OR KSA)) | Topic Search (TS) searches Title, Abstract, Author Keywords |
| EBSCO (Academic Search Complete / CINAHL) | (tuberculosis OR "Mycobacterium tuberculosis" OR TB) AND (prevalence OR epidemiology OR incidence) AND ("healthcare worker" OR HCW OR nurse OR physician) AND ("Saudi Arabia" OR "Kingdom of Saudi Arabia" OR KSA) | Applied across ASC and CINAHL simultaneously |
| Google Scholar | "tuberculosis" AND "prevalence" AND "healthcare workers" AND "Saudi Arabia" | First 200 results screened manually for relevance |
| Additional Google Scholar combinations | 1. "latent tuberculosis" AND "HCWs" AND "Saudi Arabia" 2. "tuberculosis infection" AND "health personnel" AND "Saudi Arabia" | Used to broaden coverage and capture potentially missed studies |
