# Supplementary material for: Prevalence of latent tuberculosis infection among healthcare workers in Saudi Arabia: a systematic review and meta-analysis
Source: BMC Public Health. 2026 Jun 1;26:2339. doi: 10.1186/s12889-026-27751-0 (PMC13466511; doi:10.1186/s12889-026-27751-0)

Study or Subgroup	Physician Events	Physician Total	Nurse Events	Nurse Total	Weight	Odds Ratio M-H, Random, 95% CI	Year
Abbas et al. 2010	64	429	150	1164	20.3%	1.19 [0.86, 1.63]	2010
Nabil A. 2015	7	36	21	73	13.0%	0.60 [0.23, 1.57]	2015
Al Hajoj, 5 May 2016	11	44	260	849	16.1%	0.76 [0.38, 1.52]	2016
Bukhary, 5 April. 2018	20	100	12	160	15.3%	3.08 [1.43, 6.63]	2018
Almohaya, 4 Jun. 2020	18	123	514	1745	18.3%	0.41 [0.25, 0.68]	2020
Almugti, 13 Sep.2022	20	142	32	132	17.1%	0.51 [0.28, 0.95]	2022
**Total (95% CI)**		**874**		**4123**	**100.0%**	**0.83 [0.48, 1.44]**	
Total events	140		989				

Heterogeneity: $Tau^2 = 0.36$; $Chi^2 = 26.14$, df = 5 ($P < 0.0001$); $I^2 = 81\%$

Test for overall effect: Z = 0.65 (P = 0.52)

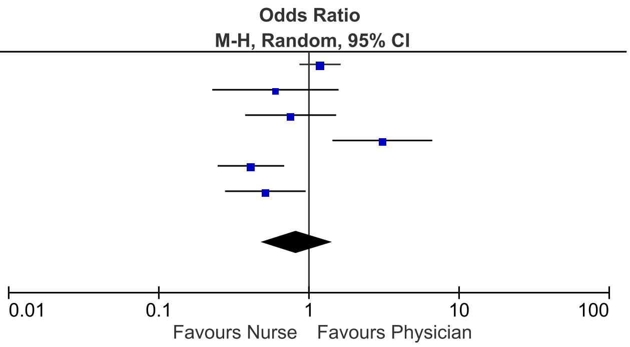

Supplement: Supplementary file 1 [file 12889_2026_27751_MOESM1_ESM.zip › Supplemntary Figure 1.pdf]
