# Supplementary material for: Prevalence of latent tuberculosis infection among healthcare workers in Saudi Arabia: a systematic review and meta-analysis
Source: BMC Public Health. 2026 Jun 1;26:2339. doi: 10.1186/s12889-026-27751-0 (PMC13466511; doi:10.1186/s12889-026-27751-0)

Study or Subgroup	Physicians Events	Physicians Total	Technicians Events	Technicians Total	Weight	Odds Ratio M-H, Random, 95% CI	Year
Abbas et al. 2010	64	429	41	423	31.9%	1.63 [1.08, 2.48]	2010
Nabil A. 2015	7	36	5	29	13.3%	1.16 [0.33, 4.12]	2015
Bukhary, 5 April. 2018	20	100	4	50	15.3%	2.88 [0.93, 8.93]	2018
Almohaya, 4 Jun. 2020	18	123	22	92	24.6%	0.55 [0.27, 1.09]	2020
Almugti, 13 Sep.2022	20	142	4	32	15.0%	1.15 [0.36, 3.62]	2022
**Total (95% CI)**		**830**		**626**	**100.0%**	**1.23 [0.70, 2.18]**	
Total events	129		76				

Heterogeneity: Tau² = 0.22; Chi² = 9.16, df = 4 (P = 0.06); I² = 56%

Test for overall effect: Z = 0.72 (P = 0.47)

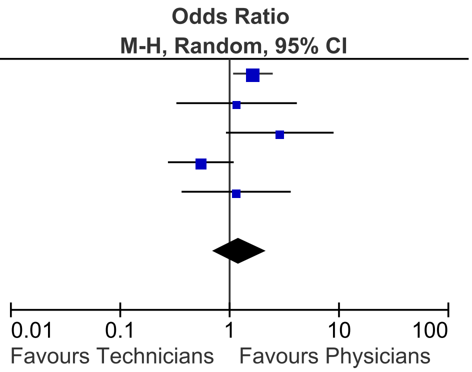

Supplement: Supplementary file 1 [file 12889_2026_27751_MOESM1_ESM.zip › Supplemntary Figure 2.pdf]
