# Supplementary material for: Prevalence of latent tuberculosis infection among healthcare workers in Saudi Arabia: a systematic review and meta-analysis
Source: BMC Public Health. 2026 Jun 1;26:2339. doi: 10.1186/s12889-026-27751-0 (PMC13466511; doi:10.1186/s12889-026-27751-0)

Study or Subgroup	Male Events	Male Total	Female Events	Female Total	Weight	Odds Ratio M-H, Random, 95% CI	Year
Abbas et al. 2010	121	1180	170	1470	14.1%	0.87 [0.68, 1.12]	2010
Balkhy. December 13, 2013	20	38	43	85	7.9%	1.09 [0.50, 2.33]	2013
Nabil A. 2015	20	88	27	120	9.0%	1.01 [0.52, 1.95]	2015
Al Hajoj, 5 May 2016	132	349	267	743	13.9%	1.08 [0.83, 1.41]	2016
Bukhary, 5 April. 2018	46	348	10	172	8.4%	2.47 [1.21, 5.02]	2018
Almohaya, 4 Jun. 2020	162	875	571	2149	14.6%	0.63 [0.52, 0.76]	2020
Almugti, 13 Sep.2022	24	176	36	148	10.1%	0.49 [0.28, 0.87]	2022
Ouban, 17 Jun.2023	30	150	36	411	10.6%	2.60 [1.54, 4.41]	2023
Aldhawyan, 1 Feb. 2024	28	92	238	679	11.3%	0.81 [0.51, 1.30]	2024
**Total (95% CI)**		**3296**		**5977**	**100.0%**	**1.01 [0.75, 1.38]**	
Total events	583		1398				

Heterogeneity: Tau² = 0.16; Chi² = 42.77, df = 8 (P < 0.00001); I² = 81%

Test for overall effect: Z = 0.09 (P = 0.93)

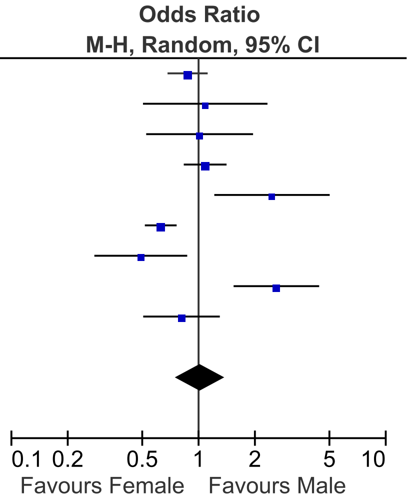

Supplement: Supplementary file 1 [file 12889_2026_27751_MOESM1_ESM.zip › Supplemntary Figure 3.pdf]
