# Supplementary material for: Prevalence of latent tuberculosis infection among healthcare workers in Saudi Arabia: a systematic review and meta-analysis
Source: BMC Public Health. 2026 Jun 1;26:2339. doi: 10.1186/s12889-026-27751-0 (PMC13466511; doi:10.1186/s12889-026-27751-0)

Study or Subgroup	prevalence	SE	Weight	prevalence IV, Random, 95% CI	Year
**2.2.1 age group >30**					
Alshahrani, 17 Jun.2023	0.0588	0.0165	6.5%	0.06 [0.03, 0.09]	2023
Almugti, 13 Sep.2022	0.303	0.04	5.7%	0.30 [0.22, 0.38]	2022
Almohaya, 4 Jun. 2020	0.2079	0.0155	6.5%	0.21 [0.18, 0.24]	2020
Al Hajoj, 5 May 2016	0.1591	0.0276	6.2%	0.16 [0.11, 0.21]	2016
Nabil A. 2015	0.1918	0.0461	5.4%	0.19 [0.10, 0.28]	2015
Abbas et al. 2010	0.0553	0.0057	6.7%	0.06 [0.04, 0.07]	2010
**Subtotal (95% CI)**			**37.0%**	**0.16 [0.08, 0.23]**	
Heterogeneity: $Tau^2 = 0.01$; $Chi^2 = 133.69$, df = 5 ($P < 0.00001$); $I^2 = 96\%$					
Test for overall effect: $Z = 4.24$ ($P < 0.0001$)					
**2.2.2 age group 30 - 49**					
Alshahrani, 17 Jun.2023	0.1276	0.0196	6.4%	0.13 [0.09, 0.17]	2023
Almugti, 13 Sep.2022	0.1471	0.0304	6.1%	0.15 [0.09, 0.21]	2022
Almohaya, 4 Jun. 2020	0.2405	0.0094	6.6%	0.24 [0.22, 0.26]	2020
Al Hajoj, 5 May 2016	0.1434	0.0207	6.4%	0.14 [0.10, 0.18]	2016
Nabil A. 2015	0.2414	0.0397	5.7%	0.24 [0.16, 0.32]	2015
Abbas et al. 2010	0.1848	0.0127	6.6%	0.18 [0.16, 0.21]	2010
**Subtotal (95% CI)**			**37.8%**	**0.18 [0.14, 0.22]**	
Heterogeneity: $Tau^2 = 0.00$; $Chi^2 = 45.23$, df = 5 ($P < 0.00001$); $I^2 = 89\%$					
Test for overall effect: $Z = 8.15$ ($P < 0.00001$)					
**2.2.3 age group > 50**					
Alshahrani, 17 Jun.2023	0.2537	0.0532	5.1%	0.25 [0.15, 0.36]	2023
Almohaya, 4 Jun. 2020	0.338	0.0279	6.2%	0.34 [0.28, 0.39]	2020
Al Hajoj, 5 May 2016	0.0976	0.0463	5.4%	0.10 [0.01, 0.19]	2016
Nabil A. 2015	0.2632	0.101	3.2%	0.26 [0.07, 0.46]	2015
Abbas et al. 2010	0.3256	0.0505	5.2%	0.33 [0.23, 0.42]	2010
**Subtotal (95% CI)**			**25.1%**	**0.26 [0.16, 0.36]**	
Heterogeneity: $Tau^2 = 0.01$; $Chi^2 = 20.92$, df = 4 ($P = 0.0003$); $I^2 = 81\%$					
Test for overall effect: $Z = 5.10$ ($P < 0.00001$)					
**Total (95% CI)**			**100.0%**	**0.19 [0.14, 0.24]**	
Heterogeneity: $Tau^2 = 0.01$; $Chi^2 = 480.88$, df = 16 ($P < 0.00001$); $I^2 = 97\%$					
Test for overall effect: $Z = 7.72$ ($P < 0.00001$)					
Test for subgroup differences: $Chi^2 = 281.04$, df = 2 ($P < 0.00001$), $I^2 = 99.3\%$					

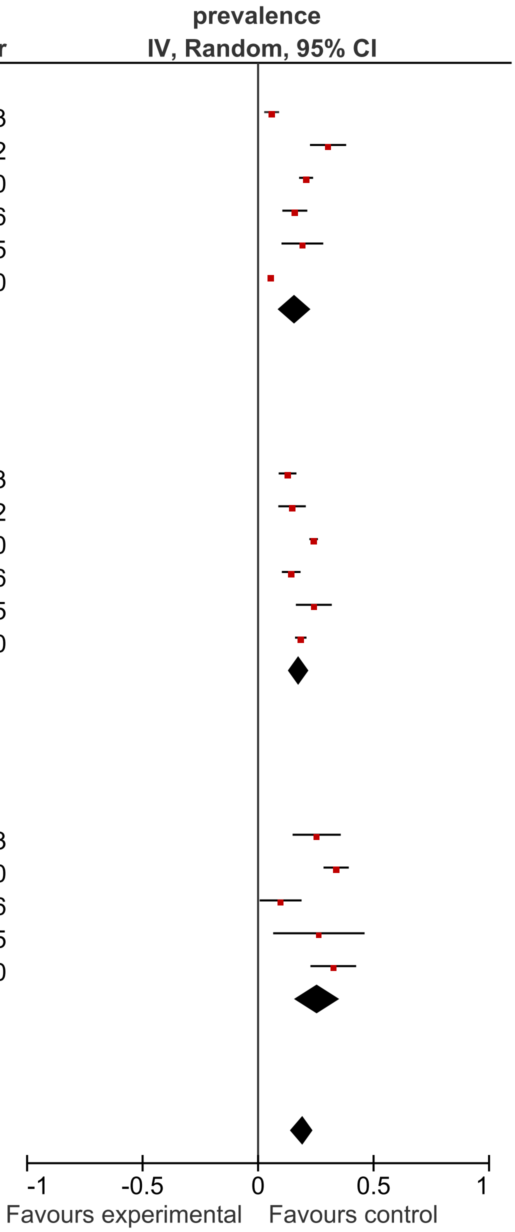

Supplement: Supplementary file 1 [file 12889_2026_27751_MOESM1_ESM.zip › Supplemntary Figure 4.pdf]
