# Supplementary material for: Prevalence of latent tuberculosis infection among healthcare workers in Saudi Arabia: a systematic review and meta-analysis
Source: BMC Public Health. 2026 Jun 1;26:2339. doi: 10.1186/s12889-026-27751-0 (PMC13466511; doi:10.1186/s12889-026-27751-0)
Supplement: Supplementary file 1 [file 12889_2026_27751_MOESM1_ESM.zip › Supplemntary Figure 5.pdf]

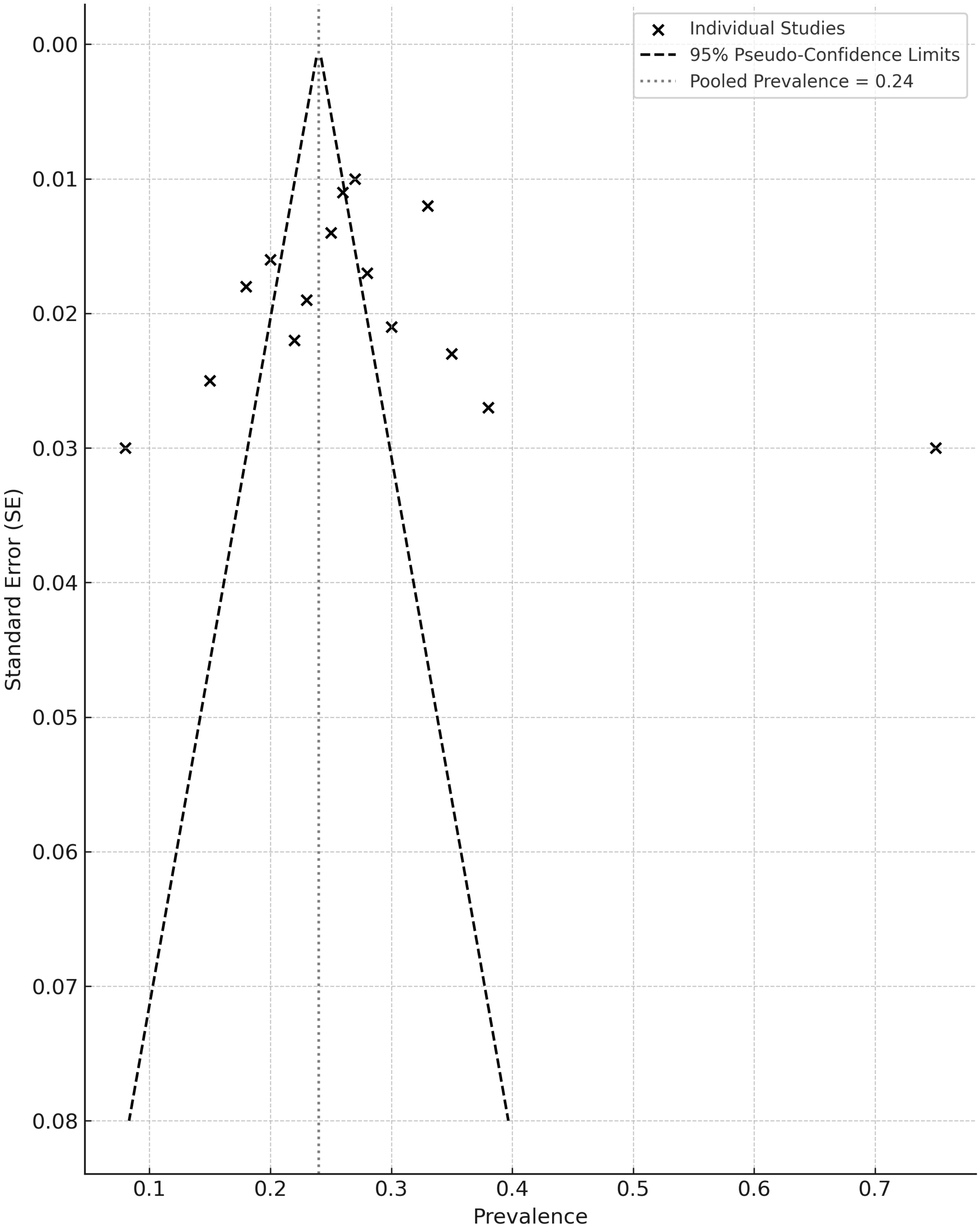

Individual Studies
95% Pseudo-Confidence Limits
Pooled Prevalence = 0.24
0.00
0.01
0.02
0.03
0.04
0.05
0.06
0.07
0.08
Standard Error (SE)
0.1
0.2
0.3
0.4
0.5
0.6
0.7
Prevalence
